# First Trimester Pregnancy Loss and the Expression of Alternatively Spliced NKp30 Isoforms in Maternal Blood and Placental Tissue

**DOI:** 10.3389/fimmu.2015.00189

**Published:** 2015-06-01

**Authors:** Avishai Shemesh, Dan Tirosh, Eyal Sheiner, Neta Benshalom-Tirosh, Michael Brusilovsky, Rotem Segev, Benyamin Rosental, Angel Porgador

**Affiliations:** ^1^The Shraga Segal Department of Microbiology, Immunology and Genetics, Faculty of Health Sciences, Ben-Gurion University of the Negev, Beer Sheva, Israel; ^2^National Institute for Biotechnology in the Negev, Ben-Gurion University of the Negev, Beer Sheva, Israel; ^3^Department of Obstetrics and Gynecology, Faculty of Health Sciences, Soroka University Medical Center, Ben-Gurion University of the Negev, Beer Sheva, Israel; ^4^Institute for Stem Cell Biology and Regenerative Medicine, Hopkins Marine Station, Stanford University School of Medicine, Stanford, CA, USA

**Keywords:** isoforms, natural killer, NCR, NKp30, pregnancy loss

## Abstract

**Capsule:** We observed that first trimester pregnancy loss is associated with an altered expression profile of the three isoforms of the NK receptor NKp30 expressed by NKs in PBMC and placental tissue.

In this study, we aimed to investigate whether first trimester pregnancy loss is associated with differences in expression of NKp30 splice variants (isoforms) in maternal peripheral blood or placental tissue. We conducted a prospective case–control study; a total of 33 women undergoing dilation and curettage due to first trimester pregnancy loss were further subdivided into groups with sporadic or recurrent pregnancy loss. The control group comprises women undergoing elective termination of pregnancy. The qPCR approach was employed to assess the relative expression of NKp30 isoforms as well as the total expression of NKp30 and NKp46 receptors between the selected groups. Results show that in both PBMC and placental tissue, NKp46 and NKp30 expressions were mildly elevated in the pregnancy loss groups compared with the elective group. In particular, NKp46 elevation was significant. Moreover, expression analysis of NKp30 isoforms manifested a different profile between PBMC and the placenta. NKp30-a and NKp30-b isoforms in the placental tissue, but not in PBMC, showed a significant increase in the pregnancy loss groups compared with the elective group. Placental expression of NKp30 activating isoforms-a and -b in the pregnancy loss groups was negatively correlated with PLGF expression. By contrast, placental expression of these isoforms in the elective group was positively correlated with TNFα, IL-10, and VEGF-A expression. The altered expression of NKp30 activating isoforms in placental tissue from patients with pregnancy loss compared to the elective group and the different correlations with cytokine expression point to the involvement of NKp30-mediated function in pregnancy loss.

## Introduction

Miscarriage, defined as the spontaneous loss of a fetus up to 20 weeks of gestation, is the most common complication of pregnancy. The prevalence of miscarriage is around 15% in clinically proven pregnancies, and up to 30% in “chemical pregnancies” (1–4). Recurrent pregnancy loss (RPL) is a term classically used in cases of three or more spontaneous miscarriages, with a prevalence of 1% of couples attempting to conceive. In recent years, the term was redefined to include two or more miscarriages, resulting in a prevalence of 3–5% of these couples (5–7). This change in definition is due to evidence showing that the risk of recurrence after two pregnancy losses is around 30%, similar to the risk of recurrence after three or more pregnancy losses (1, 3, 8, 9). Different etiologies have been suggested for miscarriages, including anatomic, genetic, endocrine, and immune disorders, as well as various infections and environmental factors. Workup studies aimed at detecting these etiologies are carried out after two or three pregnancy losses (especially in older, nulliparous women). Only in about 50% of these cases is an etiology recognized; the other cases are either of unknown etiology or thought to result from a combination of several etiologies (10–12).

NKp30 is a 30-kDa triggering receptor expressed on resting and activated natural killer (NK) cells (13). This receptor belongs to a family known as natural cytotoxicity receptors (NCRs) that include NKp46 and NKp44 (14). El Costa et al. showed that NKp30 and NKp46 on decidual NK (dNK) cells have a differential role in the early stage of pregnancy (15). Activation of NKp30 on dNK by mAb specific engagement triggers the synthesis and secretion of various cytokines. Moreover, it was shown that adherent decidual cells are recognized by NKp30–hFc fusion (16, 17).

Recently, it was shown that the human NKp30-encoding gene (NCR3) is transcribed in six different splice variants, among which the most highly expressed are NKp30-a, NKp30-b, and NKp30-c (18). Zitvogel et al. have previously published that the status and ratio of these isoforms predict the clinical outcome of patients with gastrointestinal stromal tumor (GIST). Preferential expression of the immunosuppressive NKp30-c isoform was associated with poor prognosis in these patients (19). In an additional study, the same group investigated NKp30-a and NKp30-c isoform ratios in untreated HIV-1-infected patients (18).

The reported function of NKp30 in pregnancy and pregnancy disorders and the differential expression of NKp30 activating and suppressive isoforms in cancer and HIV motivated the current study. We compared (i) patients with RPLs (>2 spontaneous miscarriages), (ii) patients with sporadic pregnancy loss (≤2 spontaneous miscarriages), and (iii) patients undergoing elective termination of pregnancy (TOP) in the first trimester due to medical or social considerations or fetal malformations as a control group. The aims of this prospective case–control study were to investigate whether first trimester pregnancy losses are associated with (A) differences in mRNA expression of the expression profile of NKp30 isoforms, as well as the total NKp30 and NKp46 expression in maternal blood and placental tissue; and (B) the correlation of NKp30, NKp46, and isoform expression with early pregnancy-related cytokines.

## Materials and Methods

### Study design and population

A single center prospective case–control study was conducted in the Department of Obstetrics and Gynecology at the Soroka University Medical Center, Beer Sheva, Israel in collaboration with the Clinical Biochemistry Laboratory at Ben-Gurion University. The study was approved by the ethical institutional review board (in accordance with the Helsinki declaration, Approval number: 0267-12-SOR).

The study population comprises women who presented to the Soroka Medical Center for dilation and curettage (D&C) during the first trimester of pregnancy (<14 weeks of gestation), who consented to participate in the study. The combined study group comprises women undergoing D&C due to first trimester pregnancy loss, who were subdivided into groups with sporadic or RPL (>2 previous spontaneous miscarriages). The control group comprises women undergoing elective TOP. Women with known uterine anomalies, multiple gestations, and women undergoing elective curettage who had a history of ≥2 spontaneous pregnancy loss were excluded from the study. The PBMC samples were elective *n* = 10, sporadic *n* = 16, recurrent *n* = 7, and Sp + Re *n* = 23, and the placental tissue samples were elective *n* = 7, sporadic *n* = 16, recurrent *n* = 7, and Sp + Re *n* = 23.

### Blood samples, PBMC isolation, and placental tissue samples

Collection of blood samples was performed using a BD Vacutainer^®^ CPT™ Cell Preparation Tube with sodium heparin (REF 362753). PBMC samples were isolated according to the manufacturer’s instructions. Placental tissue samples were collected from the retrieved specimen, just after curettage, and placed in sterile ice-cold 1× PBS until RNA extraction was performed.

### RNA extraction and reverse transcription PCR

Total RNA was extracted from fresh placenta tissue and isolated PBMC using the RNeasy^®^ Mini Kit (cat# 74104, Qiagen Ltd.) according to the manufacturer’s instructions and was stored at −80°C until further use. RNA concentration and purity were assessed by NanoDrop (ND1000 v3.7.1; NanoDrop Technologies, DE, USA). Reverse transcription PCR (RT-PCR) was performed using a High Capacity cDNA Reverse Transcription Kit (cat# 4368814, Applied Biosystems). cDNA synthesis was performed according to the manufacturer’s instructions using 1 μg of total RNA and was performed for all of the samples at the same time. cDNA samples were stored at −20°C until further use.

### Real time PCR and primer set efficiencies

qPCR analysis was performed to determine and compare the expression of target genes using the Power SYBR^®^ Green PCR Master Mix (cat# 4367659, Applied Biosystems). Twenty microliters of reactions were set-up in duplicate for each sample according to the manufacturer’s instructions. Each reaction contained 30 ng of cDNA. qPCR experiments were performed using the Applied Biosystems 7500 Real Time PCR system, software v2.0.5. Reaction cycling protocol was 95°C for 10 min, then 40 cycles with a 2-step program (95°C for 15s, 60°C for 1 min). Collection of data was performed during the annealing step. Expression levels of NKp46, NKp30, NKp30-a, NKp30-b, NKp30-c, IFNγ, TNFα, IL-10, PLGF, and VEGF (target genes) were normalized to β-actin (reference gene). For each gene, all samples per tissue (blood or placenta) were checked in the same plate. Plate normalization was performed by checking the β-actin of two fixed samples in each plate and adjusting Ct values accordingly. Calculation of gene expression was performed using the 2^−ΔCt^ = 2^−(Target gene Ct) − (Reference gene Ct)^ method. Ratio analysis of NKp30 isoforms was calculated using the NKp30_x_/NKp30_y_ = 2^(ΔΔCt NKp30y−ΔΔCt NKp30x)^ formula, as previously described (18). Primer efficiencies were tested on cDNA pools, obtained by taking 30 ng from each cDNA sample, originated from PBMC or placental tissue samples (cDNA pools were serial diluted five times (1:10 dilutions) and were used as templates).

*NKp46*: Fw primer: 5′-GTGACCACAGCCCACCGAG-′3, Rev-primer: 5′-CTCAATGTCGCCTGTGACCAG-′3, (eff%: PBMC: 103.787%, Placenta: 104.432%), *NKp30*: Fw primer: 5′-CATGGTCCATCCAGGATCC-′3, Rev primer: 5′-GTTCCATTCCTCACCTCCTTC-′3, (eff%:PBMC: 99.125%, Placenta: 100.205%), *NKp30-a*: Fw primer: 5′-GGTGGTGGAGAAAGAACATC-′3, Rev primer: 5′-CTTTCCAGGTCAGACATTTGC-′3, (eff%: PBMC: 102.924%, Placenta: 98.568%), *NKp30-b*: Fw primer: 5′-GGTGGTGGAGAAAGAACATC-′3, Rev primer: 5′-GAGAGTAGATTTGGCATATTTGC-′3, (eff%:PBMC: 99.486%, Placenta: 98.387%), *NKp30-c*: Fw primer: 5′-GGTGGTGGAGAAAGAACATC-′3, Rev primer: 5′-CATGTGACAGTGGCATTTGC-′3, (eff%:PBMC: 98.145%, Placenta: 101.560%), β*-actin*: Fw primer: 5′-GCATTGTTACCAACTGGGAC-′3, Rev primer: 5′-GGTCTCAAACATGATCTGGG-′3, (eff%:PBMC: 99.865%, Placenta: 103.251%), *IFN*γ: Fw primer: 5′-GCCAGGACCCATATGTAAAAGA-′3, Rev Primer: 5′-TTCTGTCACTCTCCTCTTTCCAA-′3, (eff%:PBMC: 99.953 5%, Placenta: 100.271%), *TNF*α: Fw primer: 5′-GACAAGCCTGTAGCCCATGT-′3, Rev Primer: 5′-TTATCTCTCAGCTCCACGCC-′3, (eff%:PBMC: 101.426 5%, Placenta: 100.384%), *IL-10*: Fw primer: 5′-TGCTGGAGGACTTTAAGGGTTA-′3, Rev Primer: 5′-ACAGGGAAGAAATCGATGACAG-′3, (eff%:PBMC: 97.45%, Placenta: 100.899%), *PLGF*: Fw primer: 5′-GATGAGCATGGTGGTTTTCC-′3, Rev Primer: 5′-AGACACAGGATGGGCTGAAC-′3, (eff%:PBMC: 101.981 5%, Placenta: 100.384%), *VEGF-A*: Fw primer: 5′-GCAGAATCATCACGAAGTGGT-′3, Rev Primer: 5′-ATCAGGGTACTCCTGGAAGATGT-′3, (eff%:PBMC: 98.751 5%, Placenta: 99.1%). Target and reference human gene sequences were taken from the National Center for Biotechnology Information (NCBI, www.ncbi.nlm.nih.gov).

### Statistical analysis

Graphics and statistical analysis were performed using GraphPad/Prism5 software. Statistical significance was tested using the two-tailed Mann–Whitney test. Correlation was tested using Spearman’s rank correlation coefficient. *p* < 0.05 was considered statistically significant.

## Results

Thirty-three women consented to participate in the study. These included 16 patients with sporadic pregnancy loss (“sporadic” group), 7 patients with recurrent (>2) pregnancy loss (“recurrent” group), and 10 patients with elective TOP (“control” group). Demographic and clinical characteristics of the three groups are summarized in Table 1. No significant differences were noted between the groups regarding maternal age or gestational age at performance of the D&C. In the control cases, three placental tissue samples were excluded from the analyses because of insufficient RNA yield or purity.

**Table 1:** **Women’s clinical histories**.

	Elective (control) (*n* = 10)	Sporadic.Ab (*n* = 16)	Recurrent^a^ (*n* = 7)
Age (years)	26.1 ± 9.58	29.29 ± 6.89	33.42 ± 6.8
Gravidity	2.3 ± 2.05	2.82 ± 1.81	6.57 ± 2.63
Parity	0.9 ± 1.28	1.58 ± 1.83	2.71 ± 1.6
Spontaneous Ab	0 ± 0	1.17 ± 0.39	4 ± 1.29
Gest. age	8.66 ± 1.42	9.88 ± 1.72	8.85 ± 1.46

*^a^Recurrent (recurrent pregnancy loss)*.

*Age, obstetrical, and infertility histories of women with elective pregnancy termination, sporadic abortion, and recurrent pregnancy losses, mean ± S.D*.

### NKp46 expression, but not NKp30 is significantly enhanced in pregnancy loss PBMC

**Figure 1:**
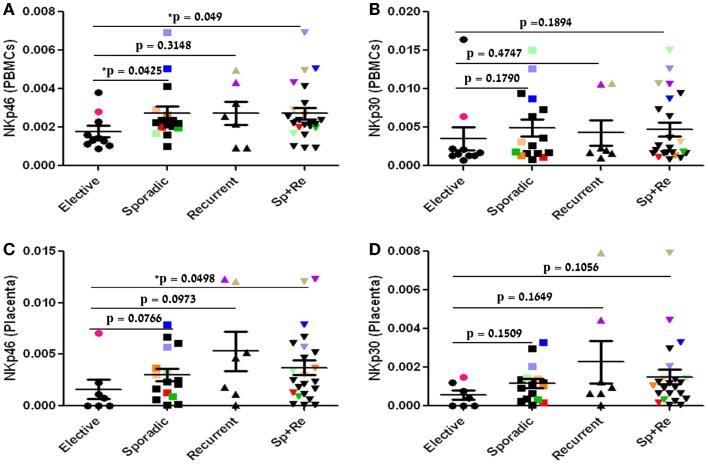
**NKp46 expression but not NKp30 is enhanced in pregnancy loss PBMC**. NKp46 and NKp30 expression analysis in PBMC and placental tissue of patients undergoing elective, sporadic, or recurrent pregnancy loss. The dot plot represents data of each patient (dotted) in each group. Bars = group mean ± SD **(A)** NKp46 mRNA (PBMC). **(B)** NKp30 mRNA (PBMC). **(C)** NKp46 mRNA (Placenta). **(D)** NKp30 mRNA (Placenta). Statistics: Mann–Whitney test, two-tailed (*p < 0.05). Ten out of the 33 individual samples are marked with colored-filled symbols (●, ■, ▲, ▼) instead of black-filled symbols for following their analysis throughout all relevant figures.

**Figure 2:**
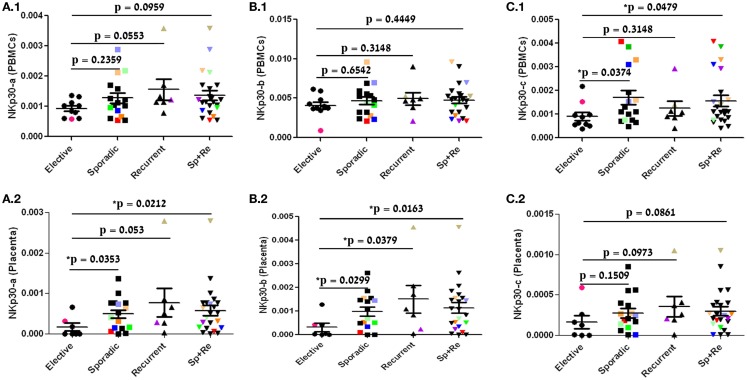
**NKp30-a and NKp30-b expression in the placental tissue is significantly elevated in sporadic and recurrent pregnancy loss**. mRNA expression analysis of NKp30 isoforms in PBMC and placenta tissue samples of patients undergoing elective, sporadic, or recurrent pregnancy loss. The dot plot represents data of each patient (dotted) in each group. Bars = group mean ± SD **(A.1)** NKp30-a mRNA (PBMC), **(A.2)** NKp30-a mRNA (Placenta), **(B.1)** NKp30-b mRNA (PBMC), **(B.2)** NKp30-b mRNA (Placenta), **(C.1)** NKp30-c mRNA (PBMC), **(C.2)** NKp30-c mRNA (Placenta), in color (demonstrated in PBMC) are individuals with a high NKp30-c profile, relative to other low NKp30-c profile samples. The NKp30-c profile in placenta tissue of the same individuals is also shown. Statistics: Mann–Whitney test, two-tailed (*p < 0.05). Ten out of the 33 individual samples are marked with colored-filled symbols (●, ■, ▲, ▼) instead of black-filled symbols for following their analysis throughout all relevant figures.

We first evaluated the expression of NKp30 and NKp46 in PBMC, as these receptors are known to be highly expressed on peripheral blood NK cells. NKp46 expression was significantly enhanced in the sporadic group compared to the control group (*p* = 0.0425, Figure 1A). NKp46 expression was also elevated in PBMC from the recurrent group compared with the control group, yet the results did not reach statistical significance. When we compared both recurrent and sporadic pregnancy loss cases as a combined group to the control group, NKp46 expression was significantly higher (*p* = 0.049, Figure 1A). NKp30 expression in PBMC samples showed the same trend in the sporadic and recurrent groups compared to the control group, yet the differences were not statistically significant (Figure 1B). We then compared NKp46 and NKp30 expression in placental tissue samples. In accordance with PBMC results, NKp46 and NKp30 expressions were enhanced in the sporadic and recurrent groups as compared to the control group, as well as for the combined group of sporadic and RPL; however, only for the combined sporadic and recurrent groups were NKp46 elevation statistically significant relative to the control group. (Figures 1C,D). Note that 10/33 individual samples are marked with colored-filled symbols instead of black-filled symbols for following their analysis throughout all relevant figures. These 10 samples were chosen based on their high expression of one NKp30 isoform (Figure 2).

### NKp30-a and NKp30-b expression in the placental tissue is significantly elevated in sporadic and recurrent pregnancy loss

NKp30 mRNA can be transcribed as three major splice variants: NKp30-a, NKp30-b, and NKp30-c (19). Although NKp30 expression did not statistically differ among pregnancy abortion groups in PBMC and placental tissue samples, we focused on the expression of NKp30-a, NKp30-b, and NKp30-c isoforms, as different NKp30 isoform profiles can lead to diverse functions of NK cells.

In accordance with total NKp30 expression, the group expression of NKp30-a and NKp30-b in PBMC was higher in the sporadic and recurrent groups compared with the control group, but this difference was not statistically significant (Figures 2A.1, B.1). The NKp30-c mean group expression in the PBMC samples was significantly elevated, which can be explained by NKp30-c profile individuals (Figure 2C.1, colored dots). In contrast to the PBMC samples, NKp30 isoform expression in the placental tissue showed a different trend. NKp30-a and NKp30-b expression was significantly elevated in the sporadic group. In the recurrent group, NKp30-a elevation was close to statistically significant, while NKp30-b elevation was statistically significant (Figures 2A.2, B.2). However, NKp30-c expression did not show any statistically significant differences among pregnancy abortion groups (Figure 2C.2, colored dots).

### NKp30 isoform ratio is different between PBMC and placenta tissue in sporadic and recurrent pregnancy loss

**Figure 3:**
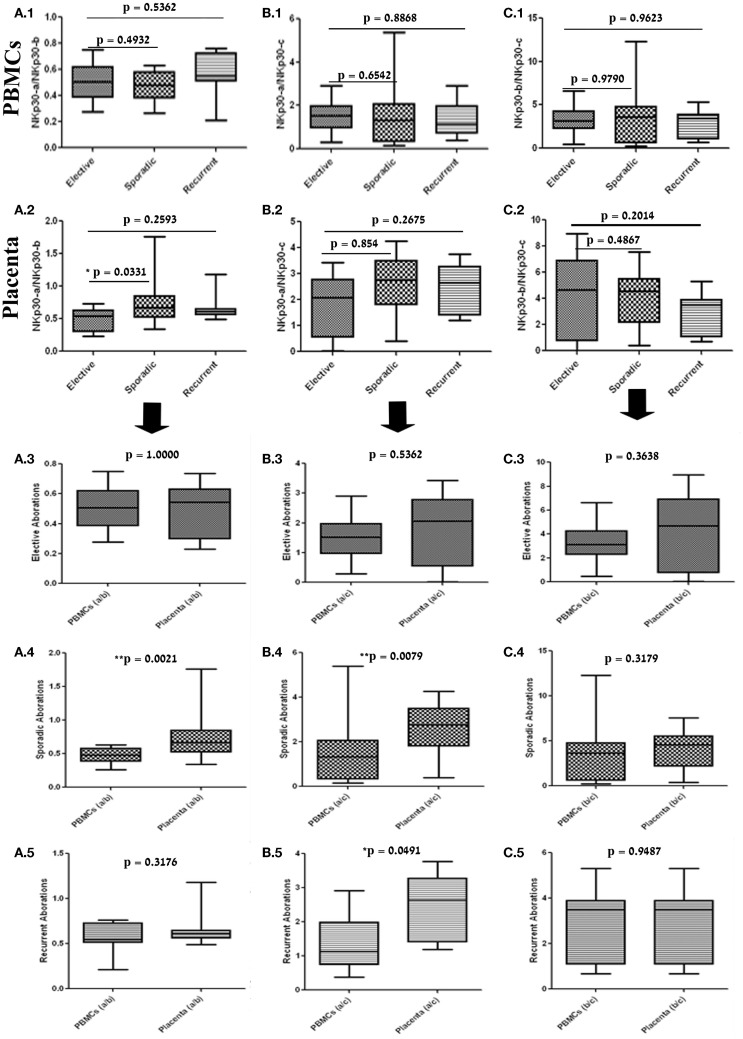
**NKp30 isoforms ratio is different between PBMC and placenta tissue in sporadic and recurrent pregnancy loss**. Ratio analysis of NKp30 isoforms per sample (median bars = median values, whiskers: Min to Max). Comparison between groups: elective, sporadic, and recurrent pregnancy loss in (1) PBMC and (2) placenta. Comparison between samples: PBMC and placenta in (3) elective, (4) sporadic, and (5) recurrent pregnancy loss. **(A.1–5)** NKp30-a/NKp30-b ratio. **(B.1–5)** NKp30-a/NKp30-c ratio. **(C.1–5)** NKp30-b/NKp30-c ratio. Statistics: Mann–Whitney test, two-tailed (*p < 0.05).

Zitvogel et al. have shown that the ratio between NKp30 isoforms can predict the clinical outcome of patients with GISTs (19). We thus studied whether a NKp30 isoform ratio could also be employed as a marker for first trimester pregnancy loss. The NKp30 isoform ratio in PBMC samples did not significantly differ between the selected groups (Figures 3A.1, B.1, C.1). The only significant change was observed in the NKp30-a/NKp30-b ratio, which was elevated in the placenta samples obtained from sporadic abortions, indicating an increase in NKp30-a relative to NKp30-b (Figures 3A.2, B.2, C.2). As we previously observed, NKp30-c profile differed in a tissue-specific manner. We then compared NKp30 isoform ratios in PBMC to placenta tissue in each pregnancy abortion group. The NKp30 isoform ratio in the elective control group did not significantly differ between PBMC and corresponding placental tissue (Figures 3A.3, B.3, C.3). In the sporadic group, NKp30-a/NKp30-b and NKp30-a/NKp30-c, but not NKp30-b/NKp30-c, ratios were significantly elevated in the placenta tissue compared to corresponding PBMC samples, indicating an increase in NKp30-a relative to NKp30-b and NKp30-c (Figures 3A.4, B.4, C.4). The same trend was seen in the recurrent group, as NKp30-a/NKp30-c was significantly elevated in the placental tissue compared to corresponding PBMC (Figures 3A.5, B.5, C.5). This data points to a tissue-specific up-regulation of NKp30-a relative to NKp30-b and NKp30-c in the placenta during first trimester pregnancy loss.

### Placental expression of NKp30-a and NKp30-b is negatively correlated with placental PLGF expression in sporadic and recurrent pregnancy loss

**Figure 4:**
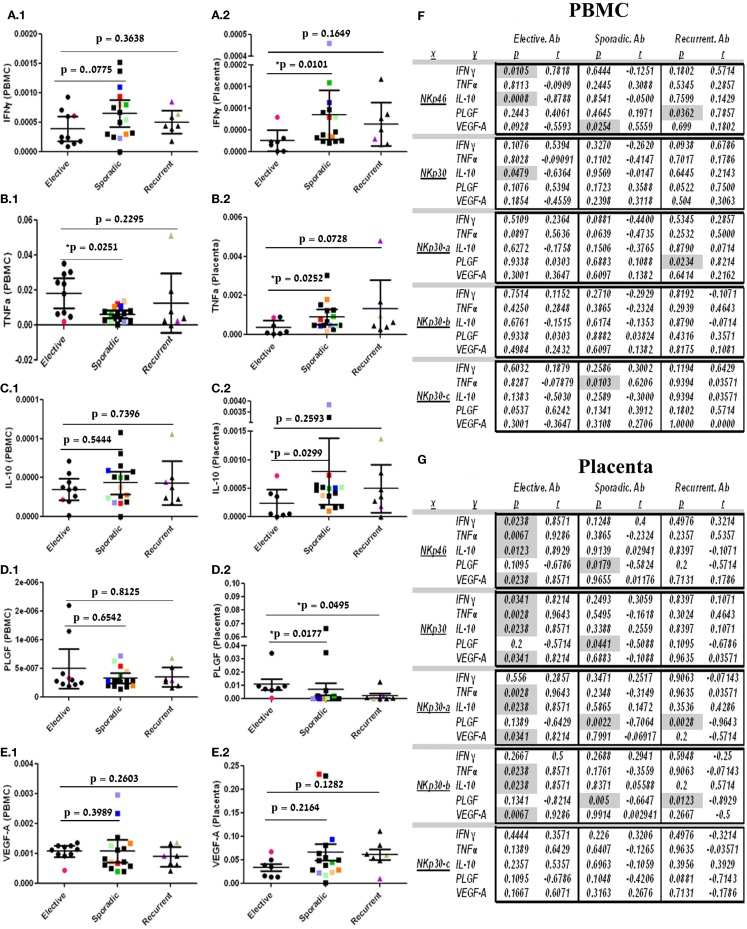
**Placental expression of NKp30-a and NKp30-b is negatively correlated with placental PLGF expression in sporadic and recurrent pregnancy loss**. IFNγ, TNFα, IL-10, PLGF, and VEGF-A mRNA expression analysis in (1) PBMC and (2) placenta tissue of patients undergoing elective, sporadic, or recurrent pregnancy loss. The dot plot represents data of each patient (dotted) in each group, Bars = group mean ± SD **(A.1,2)** IFNγ, **(B.1,2)** TNFα, **(C.1,2)** IL-10, **(D.1,2)** PLGF, and **(E.1,2)** VEGF-A. **(F)** Correlations in PBMC samples. **(G)** Correlations in placental tissue samples. Spearman’s rank correlation, (*p < 0.05). Ten out of the 33 individual samples are marked with colored-filled symbols (●, ■, ▲, ▼) instead of black-filled symbols for following their analysis throughout all relevant figures.

El Costa et al. and Hanna et al. previously showed that NKp30 engagement promote cytokine synthesis and secretion, among them IFNγ, TNFα, IL-10, PLGF, and VEGF-A, by dNK in the placental tissue (15, 16). Expression of selected cytokines did not show any significant difference among the groups in PBMC samples with the exception of TNFα, which was decreased in the sporadic group relative to the control group (Figures 4A.1, B.1, C.1, D.1, E.1). When comparing the pregnancy abortions groups in placental tissue, IFNγ, TNFα, IL-10, but not VEGF-A were significantly elevated in the sporadic but not in the recurrent group. PLGF expression was decreased in both the sporadic and recurrent abortion groups compared to the control group (Figures 4A.2, B.2, C.2, D.2, E.2). We then examined the correlation between NKp46 and NKp30, as well as NKp30 isoforms to early pregnancy-related cytokines (Figures 4F,G). Control group PBMC samples showed a positive correlation between NKp46 and IFNγ and a negative correlation between NKp46 and NKp30 to IL-10. This trend was lost in the sporadic and recurrent PBMC samples. Excluding that, a specific correlation pattern could not be seen in PBMC samples obtained from control, sporadic, and recurrent first trimester pregnancy loss cases.

In contrast to PBMC, placenta tissue samples showed a specific correlation pattern. In the elective, control group, positive correlations were seen between NKp46 and NKp30 to IFNγ, TNFα, IL-10, and VEGF-A. NKp30-a and NKp30-b kept the same correlation pattern as NKp46 and NKp30, with the exception of the correlation to IFNγ that was lost. In the sporadic placenta tissue samples, this correlation pattern was lost. However, negative correlations between PLGF and NKp46, NKp30, NKp30-a, and NKp30-b were observed. This negative correlation of NKp30 activating isoforms to PLGF was repeated in the RPL placenta samples.

## Discussion

First trimester pregnancy losses are a common complication of early pregnancy. Throughout human pregnancy, the genetically “foreign” semi-allogeneic fetus and placenta evade maternal immune responses (20). Successful pregnancy requires suppression of the mother’s immune system, enabling an immune-tolerant state. NK cells are thought to play an important role, serving as the predominant cell type in this process (21). NKp46 and NKp30 are primary activating receptors, expressed constitutively by NK cells, and their engagement can stimulate NK function (14). In the present study, we have examined in parallel the expression of NKp46 and NKp30 in both peripheral blood and placenta tissue (decidua) samples, comparing women with sporadic and recurrent spontaneous abortions to elective first trimester pregnancy terminations. Zhang et al. reported a significantly higher expression of NKp46, and a higher but not significant expression of NKp30, on CD56^dim^CD16^+^ dNK cells in women with spontaneous abortion compared with elective abortions (22). In accordance with the study by Zhang et al. in the present study, placenta NKp46 expression levels were significantly enhanced in pregnancy loss groups (combined) vs. the control elective group (Figure 1C), while placenta NKp30 expression levels were enhanced but not significantly (Figure 1D).

The report by Zhang et al. has discussed that the differences in NCR-expression correlate with a higher activity of dNK cells derived from spontaneous abortions. To better assess the NKp30 role in dNK activity in pregnancy loss, we have expanded NCR-expression studies to include NKp30 isoform expression, adding pioneer information in regard to this expression in our clinical set-up. NKp30 isoforms were reported to have opposite roles in NKp30 function, with NKp30-c bearing an inhibitory function compared to the activating functions of NKp30-a and NKp30-b (19). NKp30-a and NKp30-b were enhanced in the placenta samples of both sporadic and RPL compared to the control group (Figures 2A.2, B.2). This was unique to NKp30-a and NKp30-b in the placenta and was not observed in PBMC. Zitvogel et al. have previously published that an NKp30 isoform profile can be divided into NKp30-c or NKp30-a/b profile individuals (19). The expression of NKp30-c in the placenta did not differ significantly among the three groups. Nevertheless, in PBMC, NKp30-c mean group expression was significantly enhanced in the sporadic pregnancy loss vs. the control group (Figures 2C.1, C.2). This difference could be attributed to few patients bearing NKp30-c profile (high NKp30-c and low NKp30-a/b expression in PBMC). We marked 10 individuals expressing high levels of at least one isoform by specific coloring of their symbols (Figures 1, 2, and 4). As expected (18), patients bearing NKp30-c profile expressed low levels of the NKp30-a/b isoforms in PBMC; yet, not all placenta tissue samples corresponding to PBMC samples with NKp30-c profile showed the same profile (Figure 2, colored symbols). This phenomenon may point to a tissue-specific NKp30 isoforms profiles and will need to be addressed further.

We also investigated the NKp30 isoforms ratio profile, calculated per each patient, as a prognostic marker for the risk of abortion. We took this approach in accordance with the study by Zitvogel and colleagues regarding the prognosis of HIV patients (18). We concentrated on blood PBMC as surrogate tissue, but neither of the three mean NKp30 isoforms ratios (a/b, a/c, and b/c) was significantly different among the groups’ PBMC (Figures 3A.1, B.1, C.1). Analysis of the NKp30 isoforms ratios in placenta samples shows that the NKp30-a/NKp30-b ratio increased in the sporadic group, indicating an up-regulation in the expression of NKp30-a. The same trend was seen when comparing isoforms ratios between PBMC and placenta samples in the sporadic group and partially in the recurrent group, but not in the control group (Figures 3A.4, B.4, B.5). Because NKp30 isoforms ratios could not be subject to changes in dNK numbers, this up-regulation of NKp30-a, an activation isoforms of NKp30, may indicate extensive activation or/and function of dNK, as was reported by Zhang et al. (22).

NKp30 on dNK was reported to mediate pregnancy-related cytokine synthesis and secretion (15, 16). Fukui et al. previously reported, in PBMC, that the correlation between NCRs and selected cytokines was lost in RPL (23). Similarly in our study, in PBMC, correlation to selected cytokines was lost in sporadic and recurrent groups (Figure 4F). In the placenta tissue, a significant positive correlation was seen in the elective control group to IFNγ, TNFα, IL-10, and VEGF-A. By contrast, the sporadic and recurrent groups did not show the same correlation pattern; however, a significant negative correlation was seen to PLGF, an angiogenesis factor that was recently suggested as a marker for early pregnancy loss (Figure 4G) (24–28). These data may point out a possible positive role of NKp30-a and NKp30-b in the promotion of early pregnancy placenta development that is disrupted in early pregnancy loss.

Furthermore, we showed that NKp30-a and NKp30-b but not NKp30-c were up-regulated in sporadic and recurrent placenta samples. Hanna et al. showed that adherent decidual cells express unknown NKp30 ligands (NKp30L), which in turn may mediate cytokine secretion through NKp30, as was shown by El Costa et al. (15, 16). It may be that this up-regulation of activation isoforms of NKp30 is a compensatory process for a lack of NKp30–NKp30L recognition, which in turn damages trophoblast invasion, marked by a low expression of PLGF; yet, this hypothesis still needs to be examined.

Overall, our observations correlate with previous publications regarding the enhancement of placenta NKp46 expression during spontaneous pregnancy loss. In addition, our results shed new light on the contribution of NKp30 by demonstrating (i) changes in NKp30 isoform status between the peripheral blood and placenta in pregnancy loss, and (ii) changes between elective and spontaneous pregnancy loss in regard to NKp30 isoform status.

## Conflict of Interest Statement

The authors declare that the research was conducted in the absence of any commercial or financial relationships that could be construed as a potential conflict of interest.

## References

[1] Warburton D Fraser FC Spontaneous abortion risks in man: data from reproductive histories collected in a medical genetics unit. Am J Hum Genet (1964) 16:1–25. 14131871 PMC1932458

[2] Wilcox AJ Weinberg CR O’Connor JF Baird DD Schlatterer JP Canfield RE Incidence of early loss of pregnancy. N Engl J Med (1988) 319:189–94. 10.1056/NEJM198807283190401 3393170

[3] Regan L Braude PR Trembath PL. Influence of past reproductive performance on risk of spontaneous abortion. BMJ (1989) 299:541–5. 10.1136/bmj.299.6698.541 2507063 PMC1837397

[4] Bricker L Farquharson RG. Types of pregnancy loss in recurrent miscarriage: implications for research and clinical practice. Hum Reprod (2002) 17:1345–50. 10.1093/humrep/17.5.1345 11980763

[5] Stirrat G Recurrent miscarriage I: definition and epidemiology. Lancet (1990) 336:673–5. 10.1016/0140-6736(90)92159-F 1975862

[6] Rai R Regan L Recurrent miscarriage. Lancet (2006) 368:601–11. 10.1016/S0140-6736(06)69204-0 16905025

[7] Practice Committee of the American Society for Reproductive Medicine. Definitions of infertility and recurrent pregnancy loss. Fertil Steril (2008) 90(5 Suppl):S60 10.1016/j.fertnstert.2008.08.065 19007647

[8] Clifford K Rai R Regan L. Future pregnancy outcome in unexplained recurrent first trimester miscarriage. Hum Reprod (1997) 12:387–9. 10.1093/humrep/12.2.387 9070732

[9] Stephenson MD Management of recurrent early pregnancy loss. J Reprod Med (2006) 51:303–10. 16737026

[10] Christiansen OB Steffensen R Nielsen HS Varming K. Multifactorial etiology of recurrent miscarriage and its scientific and clinical implications. Gynecol Obstet Invest (2008) 66:257–67. 10.1159/000149575 18679035

[11] Toth B Jeschke U Rogenhofer N Scholz C Würfel W Thaler CJ Recurrent miscarriage: current concepts in diagnosis and treatment. J Reprod Immunol (2010) 85:25–32. 10.1016/j.jri.2009.12.006 20185181

[12] Bashiri A Gete S Mazor M Gete M Recurrent pregnancy loss – evaluation and treatment. Harefuah (2011) 150(11):852–6. 22428207

[13] Pende D Parolini S Pessino A Sivori S Augugliaro R Morelli L Identification and molecular characterization of NKp30, a novel triggering receptor involved in natural cytotoxicity mediated by human natural killer cells. J Exp Med (1999) 190:1505–16. 10.1084/jem.190.10.1505 10562324 PMC2195691

[14] Brusilovsky M Rosental B Shemesh A Appel MY Porgador A. Human NK cell recognition of target cells in the prism of natural cytotoxicity receptors and their ligands. J Immunotoxicol (2012) 9:267–74. 10.3109/1547691X.2012.675366 22524686

[15] El Costa H Casemayou A Aguerre-Girr M Rabot M Berrebi A Parant O. Critical and differential roles of NKp46- and NKp30-activating receptors expressed by uterine NK cells in early pregnancy. J Immunol (2008) 181:3009–17. 10.4049/jimmunol.181.5.3009 18713971

[16] Hanna J Goldman-Wohl D Hamani Y Avraham I Greenfield C Natanson-Yaron S Decidual NK cells regulate key developmental processes at the human fetal-maternal interface. Nat Med (2006) 12:1065–74. 10.1038/nm1452 16892062

[17] Deshmukh US Bagavant H. When killers become helpers. Sci Transl Med (2013) 5:195fs29. 10.1126/scitranslmed.3006850 23884464 PMC3955842

[18] Prada N Antoni G Commo F Rusakiewicz S Semeraro M Boufassa F Analysis of NKp30. NCR3 isoforms in untreated HIV-1-infected patients. Oncoimmunology (2013) 2(3):e23472. 10.4161/onci.23472 23802087 PMC3661172

[19] Delahaye NF Rusakiewicz S Martins I Ménard C Roux S Lyonnet L Alternatively spliced NKp30 isoforms affect the prognosis of gastrointestinal stromal tumors. Nat Med (2011) 17:700–7. 10.1038/nm.2366 21552268

[20] Warning JC McCracken SA Morris JM. A balancing act: mechanisms by which the fetus avoids rejection by the maternal immune system. Reproduction (2011) 141:715–24. 10.1530/REP-10-0360 21389077

[21] Lohse SR Farkas DK Lohse N Skouby SO Nielsen FE Lash TL. Validation of spontaneous abortion diagnoses in the Danish national registry of patients. Clin Epidemiol (2010) 2:247–50. 10.2147/CLEP.S13815 21152251 PMC2998814

[22] Zhang Y Zhao A Wang X Shi G Jin H Lin Q. Expressions of natural cytotoxicity receptors and NKG2D on decidual natural killer cells in patients having spontaneous abortions. Fertil Steril (2008) 90:1931–7. 10.1016/j.fertnstert.2007.08.009 18023431

[23] Fukui A Ntrivalas E Fukuhara R Fujii S Mizunuma H Gilman-Sachs A Correlation between natural cytotoxicity receptors and intracellular cytokine expression of peripheral blood NK cells in women with recurrent pregnancy losses and implantation failures. Am J Reprod Immunol (2009) 62:371–80. 10.1111/j.1600-0897.2009.00750.x 19821805

[24] Lash G Naruse K Innes B Robson S Searle R Bulmer J. Secretion of angiogenic growth factors by villous cytotrophoblast and extravillous trophoblast in early human pregnancy. Placenta (2010) 31:545–8. 10.1016/j.placenta.2010.02.020 20338637

[25] Zhou Y Bellingard V Feng K McMaster M Fisher SJ. Human cytotrophoblasts promote endothelial survival and vascular remodeling through secretion of Ang2, PlGF, and VEGF-C. Dev Biol (2003) 263:114–25. 10.1016/S0012-1606(03)00449-4 14568550

[26] Muttukrishna S Swer M Suri S Jamil A Calleja-Agius J Gangooly S. Soluble Flt-1 and PlGF: new markers of early pregnancy loss? PLoS One (2011) 6:e18041. 10.1371/journal.pone.0018041 21448460 PMC3063178

[27] Wallace AE Fraser R Gurung S Goulwara SS Whitley GS Johnstone AP. Increased angiogenic factor secretion by decidual natural killer cells from pregnancies with high uterine artery resistance alters trophoblast function. Hum Reprod (2014) 29:652–60. 10.1093/humrep/deu017 24522839 PMC3949498

[28] Yokota M Fukui A Funamizu A Nakamura R Kamoi M Fuchinoue K. Role of NKp46 expression in cytokine production by CD56-positive NK cells in the peripheral blood and the uterine endometrium. Am J Reprod Immunol (2013) 69:202–11. 10.1111/aji.12062 23311919

